# Quantitative determination of iron (III) in polymaltose haematinic formulations on the Ghanaian market

**DOI:** 10.1371/journal.pone.0325846

**Published:** 2025-07-02

**Authors:** Rita Okwampah, Kofi Dzorgbenyuie Bedzra, Oti Kwasi Gyamfi, James Ataki, Foster Kyei, Martin Adarkwah-Yiadom, Edem Sosu, Emmanuel Boateng

**Affiliations:** 1 Pharmacy Department, Ghana Atomic Energy Commission Hospital, Accra, Ghana; 2 Cellular and Clinical Research Centre, Radiological and Medical Sciences Research Institute, Ghana Atomic Energy Commission, Accra, Ghana; 3 Public Health Programmes, School of Public Service and Governance (SPSG), Institute of Management and Public Administration (GIMPA), Accra, Ghana; 4 Department of Biochemistry and Biotechnology, KNUST, Kumasi, Ghana; 5 Department of Molecular Biology and Biotechnology, University of Cape Coast, Cape Coast, Ghana; 6 Ghana Standards Authority, Drugs and Forensic Department, Accra, Ghana; 7 Medical Physics Department, School of Nuclear and Allied Sciences, University of Ghana, Accra, Ghana; 8 University of Professional Studies, Accra; Institute of Tropical Medicine: Instituut voor Tropische Geneeskunde, BELGIUM

## Abstract

Iron is an essential element needed by living organisms to enable their bodies to perform a variety of cellular functions and it is tightly regulated in the body systems of living organisms. One major public health issue in developing countries is the problem of iron deficiency and iron deficiency anaemia in infants, young children, and adults. In sub-Saharan Africa, Ghana is one of the very few countries implementing a universal health insurance programme and therefore, iron (III) polymaltose complex suspension has been one of the medications on the National Health Insurance Scheme (NHIS) drug list. Studies conducted on iron (III) hydroxide with polymaltose in liquid dosage forms in certain parts of the world have been sub-standard. Consequently, this study aims to determine iron (III) in pharmaceutical formulations in some selected haematinics in syrups sold on the Ghanaian market. Seventeen (17) randomly selected products were analysed. Measurements were carried out at 628nm. Results obtained indicated that the concentration of iron (III) ranged between (45.3–49.2) mg/5mL and (57.9–83.4) mg/5mL. At a 95% confidence interval, the population mean of the concentration of the haematinic was between (54 mg/5mL - 66 mg/5mL). As much as anaemia can be cured with iron supplementation, excess iron overload can be very dangerous to the human body therefore Pharmaceutical products should be analysed on a regular basis to ensure their safety and effectiveness.

## Introduction

The socio-economic development of a nation is dependent on the well-being of its citizens [1]. For a high human economic productivity, the country must place a high premium on human health [2]. The World Health Organization (WHO) in 2011 estimated that around 2 billion individuals had anaemia with iron deficiency contributing approximately 50% of all cases and approximately 300 million children suffer from the condition [3]. Women of reproductive age (528.7) million, including 32.4 million pregnant women and 496.3 million non-pregnant women, were anaemic in 2011 [4]. The death toll in 2012 was estimated to be around 1 million, which was considered to be four times more common in developing nations than the developed ones with three-quarters of deaths taking place in South-East Asia and Africa [5]. It was estimated that developing countries accounted for more than 89% of all anaemia cases and about 11 million people or more than 40% of the Ghanaian population were anaemic in 2013 [6]. Studies by the WHO in Europe of the prevalence of anaemia in 2017 was found to be 19.9% in non-pregnant women aged 15–49 and 24.5% in pregnant women, with iron deficiency being the primary cause [7]. The United States Pharmacopeia has indicated that pharmaceutical products and food supplements that contain iron are registered depending on the iron content in the dosage form [8]. However, anaemia as a nutritional deficiency has a serious negative effect on global public health and the social and economic development of both developing and developed nations [9]. Iron supplementation is considered an effective strategy to counter anaemia, and iron preparations are some of the most prescribed medications [1]. Iron deficiency is a serious threat to survival because it prevents immunological and nervous system cells from growing [10]. The WHO consistently recommends oral iron supplementation as one of the interventions that can lessen the burden of anaemia, estimating the annual physical productivity loss due to iron deficiency in low and middle-income countries to be about US$ 2.32 per capita or 0.57% of gross domestic product [3]. Iron deficiency anaemia reduces productivity at work and it affects the world’s Gross Domestic Product (GDP) by 1.3% [4]. Identification and accurate concentrations of metal ions are crucial in pharmaceutical analysis. Therefore, several techniques have been developed for the quantitative analysis and determination of iron such as spectrophotometry (atomic absorption spectroscopy and atomic emission spectroscopy), voltammetry, chromatography, capillary electrophoresis, flux injection analysis [11,12]. Iron deficiency anaemia (IDA) puts people who suffer this condition at a greater risk of losing their lives especially children under the age of five, women in their fertile years, and pregnant women who are more vulnerable to iron deficiency anaemia, with prevalence rates reaching 41.7%, 32.8%, and 40.1%, respectively [13]. There is not enough data especially in terms of geographical availability on micro-nutrient deficiencies in Ghana but iron has a deficiency prevalence of about 45.0% [6]. This requires a lot of resources from Government and State agencies to prevent the condition in their various countries and the World at large. Governments must put a lot of systems in place to help curb this condition and this includes making available iron fortified medications on the markets. These medications must contain standard doses of iron to help form the necessary haemoglobin needed on an individual basis to alleviate this condition.

Studies conducted by Vasile and colleagues on iron (III) hydroxide with polymaltose in liquid dosage forms in Moldovia indicated that the concentrations of some of the analysed samples were less than the 50 mg/5mL concentration stated by the manufacturer of these products [14]. In a study by Abualhasan and colleagues in Palestine in 2021 of iron supplements available on the Palestinian market, they indicated that 72% of the samples they tested had exceeded the standard limit and had failed the test [15]. Thus, the need for this research is to help establish if haematinics on the Ghanaian market are formulated with the standard dosages of iron to help alleviate this public health concern. Spectrophotometry was employed for this analysis due to its suitability for the assessment of iron in syrups and the non-interference of syrup excipients [16].

### Anaemia

Anaemia, worldwide, is one of the major public health issues that generally affects over half the world’s population of pre-schoolers, pregnant women, and non-pregnant women [17]. The World Health Organization’s (WHO) regional estimates for the condition in developing countries was around (47.5–67.6%) with the highest proportion originating from Africa and therefore exerting a negative socio-economic impact on the population [9]. Common causes of anaemia include gastrointestinal diseases, trauma, low dietary iron intake, and poor iron absorption [17]. The most common categorisation of anaemia is found in a variety of red cell defects, including those related to production (aplastic anaemia), maturation (megaloblastic anaemia), defects in haemoglobin synthesis (iron deficiency anaemia), genetic defects of haemoglobin maturation (thalassaemia), and the physical loss of red blood cells (haemolytic anaemia) [18]. The causes of anaemia are multifaceted and complex, and iron deficiency is the main factor contributing to the burden of about 50% of the cases worldwide [19]. The situation of anaemia as estimated by WHO was 41% for women and 27% for children worldwide with prevalence among children less than 5 years at 42.6% with South-East Asia and Africa contributing 62.3% and 53.8% respectively [20,21]. As much as anaemia can be cured with iron supplementation, excess iron overload causes liver fibrosis and cirrhosis, cancer, diabetes, cardiovascular disease, and many neuro-degenerative conditions [22].

### Iron

The most abundant, essential, and trace mineral found in the living cells of almost every organism on earth is iron [23,24] and mostly found in the mitochondria, which is the energy hub for all cell energy production [22]. As is known, iron exists in the erythrocytes (RBC) where it is used in the synthesis of Haemoglobin (the Haem compound), in muscle cells as myoglobin, in the transports of Oxygen and Carbon dioxide in the lungs and various peripheral tissues and plays an important role in blood pH regulation [25–27]. Excess iron is deposited in the liver as hemosiderin and ferritin in myoglobin and in Haem and non-Haem enzymes [28] acts as the prosthetic group in crucial biological processes, and multi-protein complexes such as the mitochondrial respiratory chain complexes [29–31]. Its abundance however is not proportional to its bioavailability [32]. Haem iron is found in animal foods such as meat, seafood, and poultry has shown bioavailability of up to 40.0% while non-haem iron in plant foods such as whole grains, nuts, seeds, legumes, leafy greens, animal flesh, and fortified foods are less-well absorbed with bioavailability of about 2–20% [30,33].

#### Iron deficiency.

The depletion of iron stores, smaller, paler red blood cells (RBCs), and low haemoglobin levels are the hallmarks of iron deficiency, which can advance to iron deficiency anaemia or persist at depleted levels without progressing with Mean Cell Volume (MCV) and Mean Cell Height (MCH) values [34,35]. Women of reproductive age due to their menstrual cycle are more prone to have an iron deficit status due to blood loss [36]. In pregnant women, deficiency in iron leads to placental hypertrophy that increases the chances of babies being born preterm or having low birth weight, or both conditions, perinatal problems, and in some cases maternal mortality [37,38]. This is also linked to delayed foetal brain maturation, paediatric cognitive impairments, and maternal depression [39]. During pregnancy, permanent iron loss is approximately 840 mg with the pregnant woman losing about 680 mg which can lead to iron deficiency anaemia [37]. Although a lack of iron in the human body will lead to iron-deficiency anaemia, excess iron can also be potentially toxic and should be tightly controlled at the cellular level [40].

#### Iron deficiency anaemia.

One of the contributing factors to iron deficiency anaemia is Malaria which causes intravascular haemolysis with subsequent loss of haemoglobin iron leading to a reduced Haemoglobin concentration as a result of the infection [41]. Among the elderly, iron deficiency anaemia has led to conditions such as cardiovascular diseases, physical functioning impairment, restricted daily activities, and depressive symptoms that have been presented at various medical and surgical facilities [42,43]. Numerous well-designed prospective studies have demonstrated that children with iron deficiency anaemia may experience motor and cognitive delays as well as mood disorders [44]. Studies conducted on pre-school children in rural Ethiopia discovered that children within the age bracket 54–60 months old with iron deficiency anaemia scored less favourably on verbal thinking tests than children without anaemia [45]. Untreated iron deficiency anaemia affects the immune system, and circulatory system due to exposure to infections, diseases, and haem essential to organs like the brain, heart, and lungs [46]. Studies conducted by Ewusie and colleagues on the levels of anaemia prevalence within the Ghanaian population among children less than 5 years were 78.4% which is a major healthcare concern [47]. Recent studies have indicated that pregnant women in their third trimester of pregnancy are at a greater risk of developing anaemia than during the first and second trimesters of pregnancy [48]. Up to 90% of women in sub-Saharan Africa became anaemic during their pregnancy [49]. Specifically, women in their reproductive age in three regions (Northern, Volta, and Central) have shown prevalence of 49%, 48%, and 47% respectively [50]. Three categories of persons are at risk of developing iron deficiency anaemia: (a) children under 5 years of age (i.e., affects their physical development and may cause irreversible damage to motion, cognitive and behavioural development) especially those in developing countries, (b) young women of reproductive age (15–49 years) who are menstruating and need twice as much iron as men of the same age and (c) pregnant and breastfeeding women [21,33]. Iron bioavailability to curb iron deficiency anaemia is achieved by supplementation with iron which is effective and inexpensive and administered orally [19].

### Iron fortification

Iron fortification of diets if properly applied is one of the effective, long-lasting, and affordable interventions that can enhance the treatment of anaemia within populations [51]. This process has been certified and regarded as one of the strategies that require long-term goals to improve iron status [52,53]. However, countries like Ghana and others in sub-Saharan Africa, Asia and the Middle East that have mandatory fortification policies but are implementing medium-term strategies will not entirely improve the iron status of their populations [53,54]. As a result of this, widespread use of oral iron supplements will be needed to improve the iron status using compounds such as iron-polymaltose complex and iron protein succinylate [55,56].

### Iron overload

Non-intentional sources of iron overload can occur from hereditary hemochromatosis, higher iron absorption from a typical diet, and repeated iron-containing chemical infusions [57]. The accumulation of excess iron will cause organ failure in the liver and heart [28]. This situation will lead to ferroportin disease also known as hemochromatosis type 4 as a result of mutations occurring in the *SLC40A1* gene associated with iron regulation, thereby causing dysregulation of hepcidin production [58]. In the brain, as a result of ageing, iron overload has been linked to Alzheimer’s disease, Parkinson’s disease, and other degenerative conditions because of mutations in the DNA caused by free radical oxygen species [59].

### Supplementation with iron polymaltose (III) complex

Iron deficiency anaemia can be reversed by supplying enough iron to normalize Haemoglobin concentrations and replenishing iron stores [39]. Oral supplementation with the use of syrups and tablets containing iron are one of the means of restoring haemoglobin levels to a steady state [60]. A variety of iron salt formulations are available for this purpose [61]. Ferrous or iron (II) salts are the most prescribed oral iron supplements and the cheapest as compared with ferric or iron (III) salts which are easily absorbed into the bloodstream resulting in a rapid increase in serum iron concentration [62]. A combination of polynuclear iron (III) oxyhydroxide core and polymaltose ligands is the most popular ferric or iron (III) formulation used for oral supplementation [63]. Gastrointestinal irritations may occur, but consistently lower rates are observed with iron (III) polymaltose complex than with ferrous or iron (II) sulphate treatment [64]. The advantage of iron polymaltose complex is a lower adverse reaction incidence in comparison with ionized iron (II) compounds such as iron (II) sulphate [14]. In sub-Saharan Africa, Ghana is one of the very few countries implementing a universal Health Insurance programme [65]. One of the medications on the National Health Insurance Scheme drug list of Ghana is Iron (III) Polymaltose Complex [66]. Elsewhere, the Ministry of Health of the Republic of Moldova directed the use of the complex of iron (III) hydroxide with polymaltose in the form of syrups, solutions, or tablets for the treatment of iron deficiency anaemia in children [67]. Ghana has also put systems in place (i.e., making available iron-fortified medications) that contain standard doses of iron to help form the necessary haemoglobin needed on an individual basis to alleviate this situation [68]. This work aims to establish if iron (III) polymaltose preparations on the Ghanaian market are formulated with the standard dosages of iron as stated on the manufacturer’s labels.

## Materials and methods

All chemicals used in this study were procured from reliable sources in Germany and China. UV/Visible Spectrophotometer (CP 83059−15, Cole Parmer Instruments Company, 625 East Hills, IL), pH- meter (Oakton^®^ pH 700 Benchtop pH Meter, Eutech Instrument, Singapore), Analytical balance (Sartorius CP64, Sartorius AG Gottingen, Germany), Water bath (GFL 1002, Gesellschaft fur Labortechnik mbH, Germany), Iron (III) polymaltose powder (% Purity: 36.54% w/w (Iron)), Methyl thymol blue (Guangdong Guanghua Sci-Tech Co., Ltd, China), Concentrated hydrochloric acid (HCl), and Concentrated nitric acid (Merck, Germany).

### Sample selection and collection

Products containing iron (III) polymaltose were randomly purchased. A list of pharmacies within the Greater Accra Region was made and with reference to products that contained polymaltose on the NHIS list of medications and approved by the Food and Drugs Authority (FDA) was selected for this study. There were eleven (11) pharmaceutical companies that stocked both locally and foreign haematinics that contained polymaltose. The systematic random sampling method was employed and twelve (12) different brands were selected. The 12 brands contained polymaltose. Out of the 12 brands, 5 brands had 2 samples each that brought the total analysed samples to 17 (i.e.,12 brands gave us 17 samples). Only the 12 brands contained the molecule of interest, and that is why they were included in the study. The manufactured date of all the products were in 2020 and were due to expire in 2024. Sampling was done in July 2022 and worked on in that same year.

### Preparation of standard calibration curve

**Fig 1:**
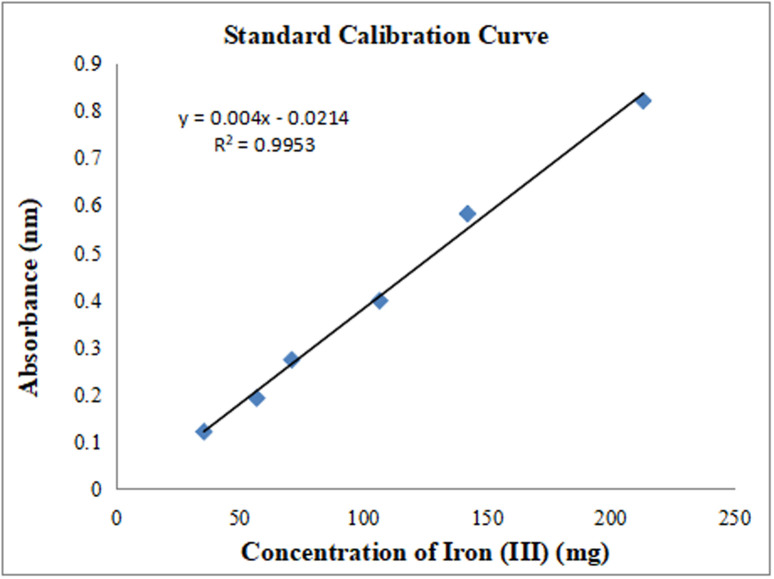
Standard calibration curve for complex Fe^3+^ MTB complex plotted through linear regression y = ax + b.

All chemicals used in the preparation of the solutions were of analytical grade. A sample stock solution of iron was prepared by weighing (2.055 g) of Iron (III) polymaltose powder (36.54% w/w) containing 0.1420g of pure iron, dissolved in 100 mL volumetric flask with double distilled water (Solution A). Serial dilutions of stock solution were prepared by diluting 0.4mL of solution with acetate buffer solution (pH 5) and topping it up to the 50 mL mark in a volumetric flask. A stock solution of Methyl Thymol Blue (MTB) was prepared by dissolving (3.2389 g) in a 100 ml volumetric flask with doubled distilled water. 0.4 mL of this solution was diluted in a 50 mL volumetric flask with acetate buffer solution (pH 5). Calibration solutions of the Fe^3+^: MTB were prepared containing equal volumes of solutions and kept in a water bath at 30°C for 30 minutes. The absorbance of the solutions was measured at 628 nm. The calibration curve was drawn using the formula y = ax + b, where y stands for absorbance and x for concentration *a* represents the slope, *b* is the intercept and *R*^*2*^ is the correlation coefficient. Within the concentration interval of (30–215) mg, the correlation coefficient was 0.9953 with its equation being y* = 0.004x – 0.0214.* The most appropriate conditions for the total reaction to occur as established by Totan and colleagues was at pH 5.0 with Acetate buffer solution (1M) and a minimum reaction time of 20–25 minutes at 30°C [12]. The average of three readings for each concentration level is represented by the absorbance and sample after following Beer’s law as depicted in Fig 1 and Table 1.

**Table 1:** Absorbance of standard iron (III) polymaltose.

Mass of Iron (g)	Mean Absorbance (628nm)
0.0355	0.121
0.0568	0.192
0.0710	0.273
0.1065	0.398
0.1420	0.582
0.2130	0.821

### Sample preparation and analysis

A sample volume of approximately 5 mL was aliquoted into a 100 mL volumetric flask containing a mixture of 1 mL concentrated HCl and 5 mL concentrated HNO_3_. Subsequently, the mixture was boiled in a water-bath for 5 minutes at 100°C to ensure complete dissolution of the sample and then allowed to cool to room temperature (25°C). Double distilled water (93 mL) was added. Approximately 0.4 mL of the resulting solution was transferred into a 50 mL volumetric flask with the addition of 49.6 mL of acetate buffer solution. These solutions were prepared daily, and the absorbance was measured at 628 nm. The regression equation generated from the standard calibration curve was used to calculate the concentration of iron in the analyzed samples [12].

## Results and discussion

In line with the objectives of this study, 12 different brands of products/samples were randomly selected as shown in Table 2.

**Table 2:** Classification of samples into origination.

Sample Source	Number
Local	9
Imported	3
Total	12

This study was conducted using the spectrophotometric method adapted from Totan and colleagues [12]. Methyl Thymol Blue (MTB) which is a sensitive but non-selective metallochromic indicator that interacts with more than twenty metal ions depending on the pH value of solution was used as described by Rasouli et al. [69]. Seventeen (17) batches of samples were obtained containing iron (III) polymaltose preparations.

## Discussion

**Fig 2:**
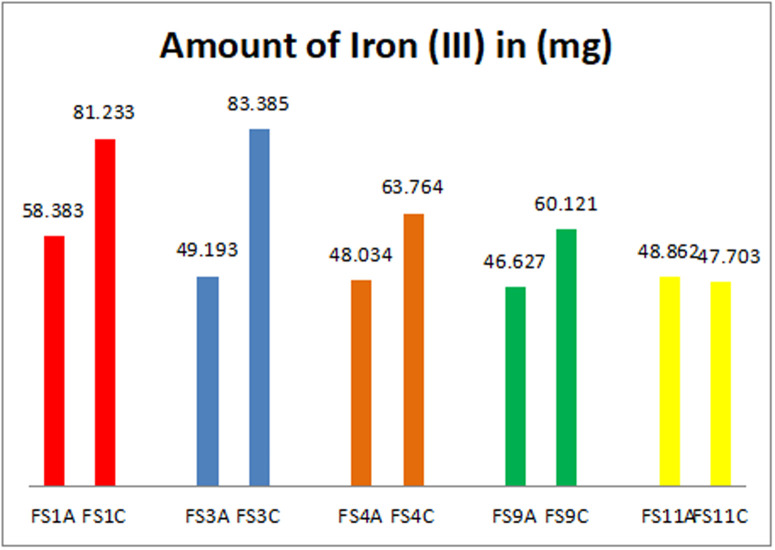
Five products that had two samples each being analyzed (first and second batch).

**Fig 3:**
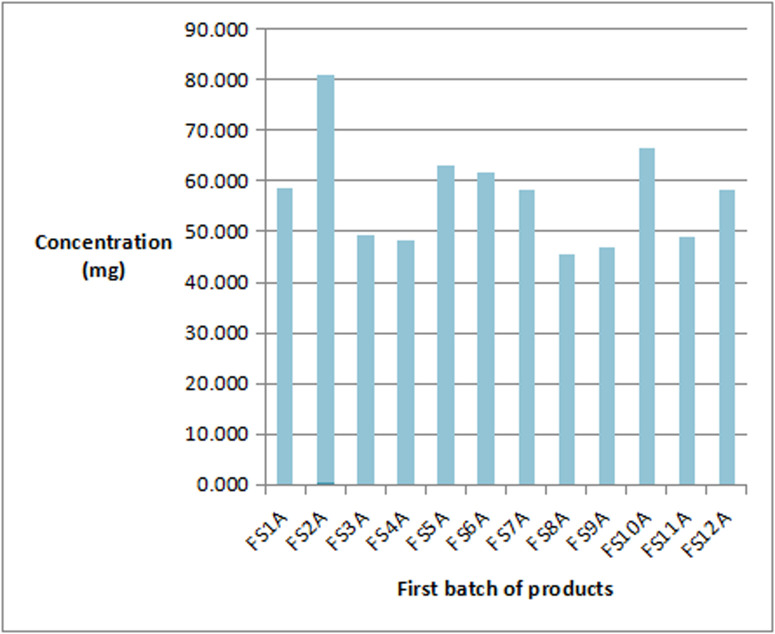
Analysis of the first batch of products from 12 different companies.

Almost all the manufacturers (15 of such) stated on their products that each 5mL volume of syrup contains 50 mg of iron (III). Two (2) others stated 32 mg/5mL and 36 mg/5mL respectively. It was realized that six (6) out of the 17 samples had concentrations ranging from (45.3–49.2) mg/5mL lower than the dosage stated on the products. The other 11 sample’s concentrations ranged from (57.88–83.38) mg/5mL which are above the stated dosage on the products. As shown in Fig 2, 5 products with two batches of samples each were analysed. In the first batch products, one product had a concentration of 58.38 mg/mL above the stated amount on the product label. Four (4) had concentrations lower than 50 mg/5mL (46.62, 48.03, 48.86, and 49.19). In their second batchs, the concentrations were higher than what the manufacturers had stated on their products (60.12, 63.76, 81.23, and 83.38) mg/5mL respectively. One sample that had a lower concentration in the first batch had a further reduction in concentration from 48.86 mg/mL to 47.70 mg/5mL which could be due to quality control procedures. Products yielding lower concentrations than the (50 mg/5mL) stated on the bottles were also highlighted in a study conducted in Moldova by Vasile and colleagues where they indicated lower concentrations of iron (III) hydroxide with polymaltose in some of their samples analysed [14]. In a study by Abualhasan and colleagues in Palestine in 2021 of iron supplements available on the Palestinian market, they indicated that 72% of the samples they tested had exceeded the standard limit and had failed the test [15]. This confirms the 65% (11/17) samples that had iron content exceeding the values stated on their labels. Further to this, they asserted that it was common practice for manufacturing companies to add more of a particular substance to products to compensate for the degradation during manufacture or shelf life of the product which was related to assertions made by Sane and collegues [70]. This practice can lead to very critical health implications for users of such products. Moreover, some formulations contained less than the stated amount of iron (III) and studies by Karelia and Buch indicated that formulations with lower than 33 mg of elemental iron (III) need to be administered more than three times a day, which can negatively affect patient compliance and increase the likelihood of side events [71]. A lot of studies around the world have shown that the claimed compositions of many supplements on the market are different from the actual quantities when tested using different analytical techniques. From the analysis six (6) out of the seventeen (17) samples had iron concentrations ranging from (45.3–49.2) mg/5mL lower than the concentration stated on the products. The concentrations of iron in the other eleven (11) samples ranged from (57.88–83.38) mg/5mL which were also higher than what manufacturers stated on the products. These haematinic products in the market were labelled as containing 50 mg/5mL of iron. The analysis however gave results ranging from 45–49 mg/5 ml and 57.9–83.4 mg/5 ml as shown in Table 3 and Fig 3. With a 95% confidence interval, the mean iron concentrations in the haematinic products are between (54 mg/5mL - 66 mg/5mL).

**Table 3:** Characteristics of samples after analysis.

Sample ID	Mean Absorbance @628nm	Estimated weight of Iron in 5 ml sample (mg)	Stdev
FS1A	0.214	58.383	0.14
FS1C	0.306	81.233	0.38
FS2A	0.303	80.570	0.25
FS3A	0.177	49.193	0.29
FS3C	0.314	83.385	0.29
FS4A	0.172	48.034	0.25
FS4C	0.235	63.764	0.38
FS5A	0.232	62.936	0.25
FS6A	0.226	61.529	0.29
FS7A	0.212	58.052	0.14
FS8A	0.161	45.302	0.25
FS9A	0.166	46.627	0.38
FS9C	0.221	60.121	0.14
FS10A	0.246	66.413	0.25
FS11A	0.175	48.862	0.29
FS11C	0.171	47.703	0.14
FS12A	0.212	57.886	0.29

## Limitations

The limitation to this study indicates that the results cannot be generalised for all haematic products on the market but it is focused on only those containing polymaltose. The reliance on data from the NHIS drug list may mean that other products not on the list but contain polymaltose might be missed during sampling. These limitations highlight the need for cautious interpretation of the results and the importance of further research to address these gaps.

## Conclusion and recommendation

Iron supplement products are mostly sold over the counter and easily accessible to all persons in need and are highly patronized by the population. This inevitably places a significant responsibility on regulatory authorities to regularly survey pharmacies and supermarkets to check and ascertain product quality. The majority of the samples had iron concentrations above 50 mg/5mL which is alarming. This calls for immediate regular control checks concerning iron content in haematinic products or syrups on the Ghanaian market. Awareness programmes regarding the use of iron-containing products should be organized for consumers, especially pregnant women, the elderly, parents, and guardians. Enforcement of good manufacturing practices and good laboratory practices must be enforced by regulatory authorities.

## Supporting information

S1 Table Classification of samples into origination. (JPG)

S2 Table Absorbance of standard iron (III) polymaltose. (JPG)

S3 Table Characteristics of samples after analysis. (JPG)

S1 File S1_ Characteristics of samples after analysi. (PDF)
