# Supplementary figures and images for: Quantitative determination of iron (III) in polymaltose haematinic formulations on the Ghanaian market
Source: PLoS One. 2025 Jul 2;20(7):e0325846. doi: 10.1371/journal.pone.0325846 (PMC12221170; doi:10.1371/journal.pone.0325846)

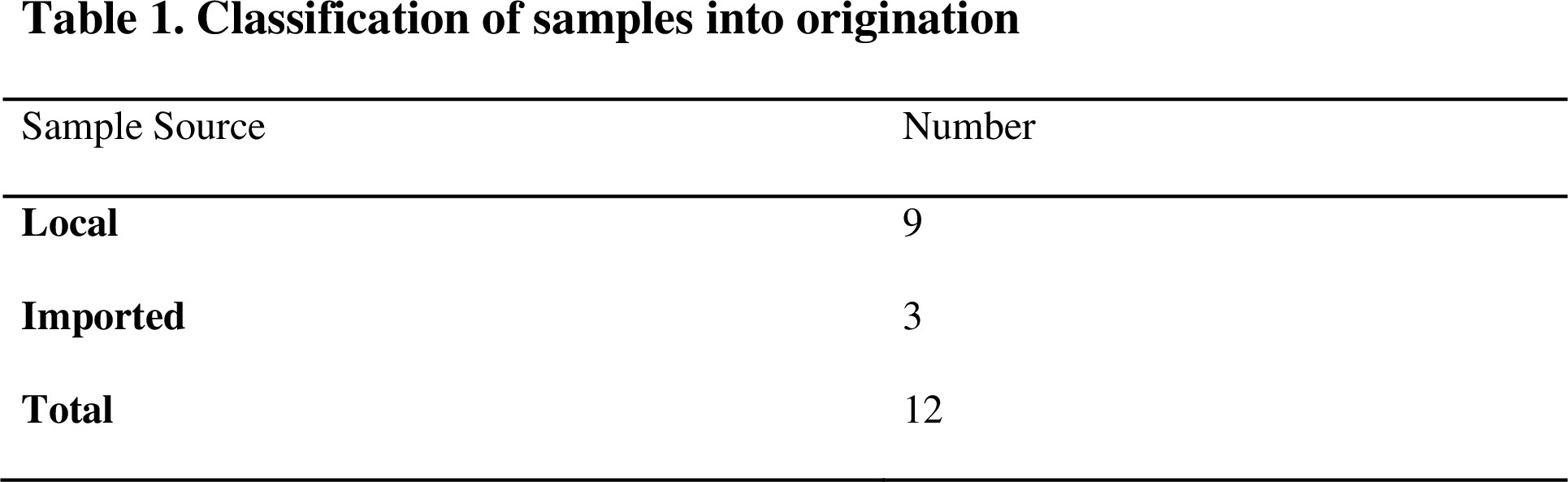

Supplement: S1 Table — (JPG) [file pone.0325846.s001.jpg]

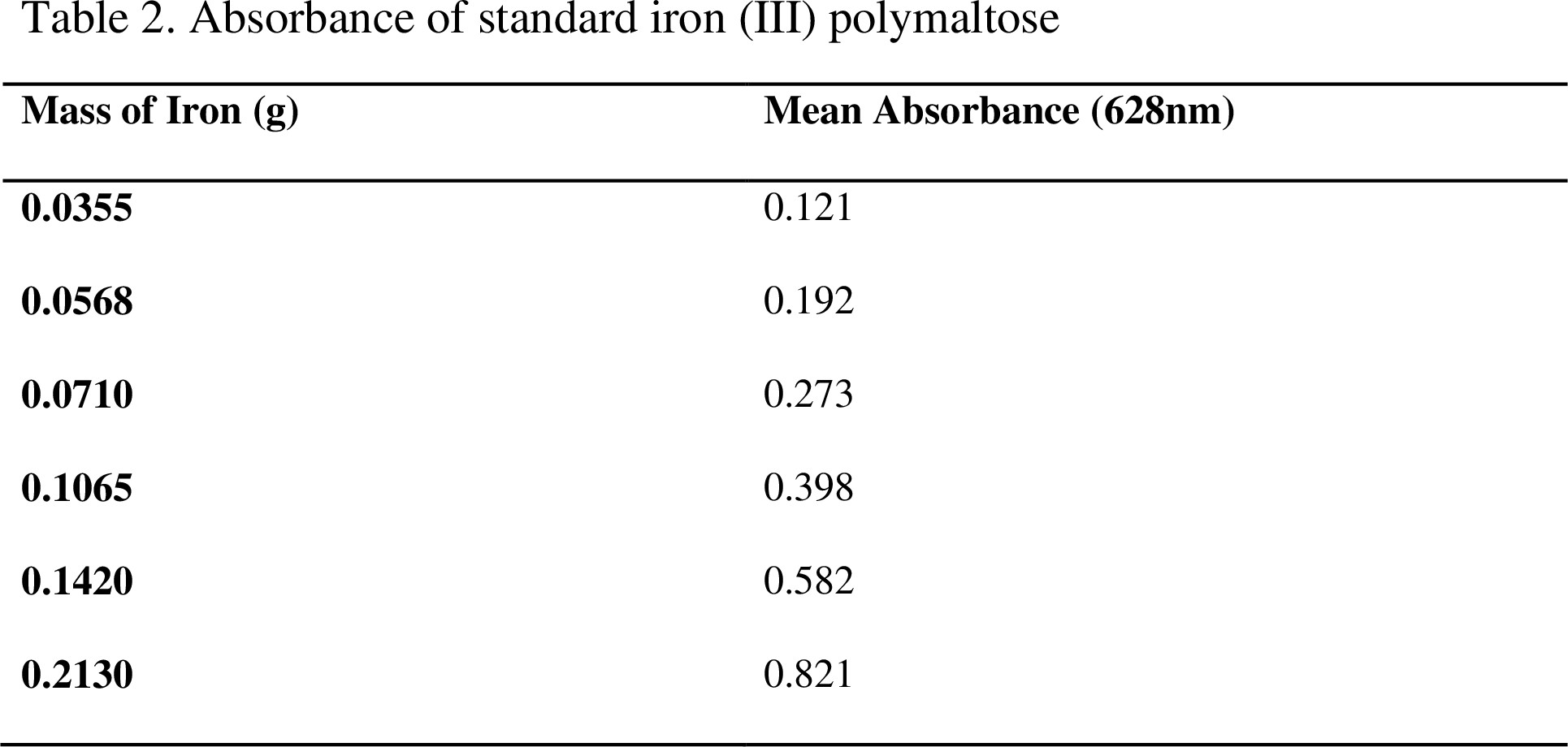

Supplement: S2 Table — (JPG) [file pone.0325846.s002.jpg]

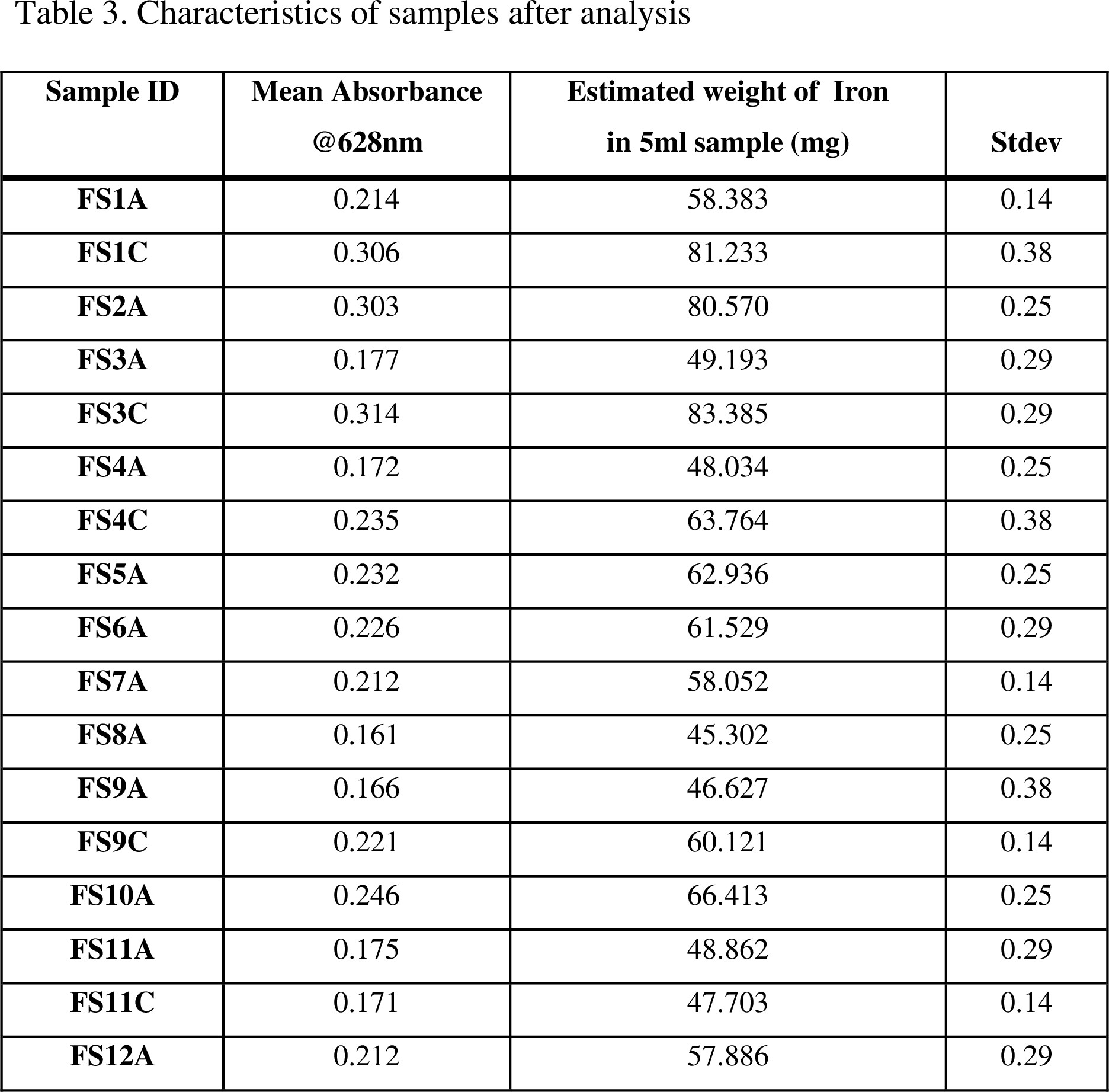

Supplement: S3 Table — (JPG) [file pone.0325846.s003.jpg]
