# Supplementary material for: Quantitative determination of iron (III) in polymaltose haematinic formulations on the Ghanaian market
Source: PLoS One. 2025 Jul 2;20(7):e0325846. doi: 10.1371/journal.pone.0325846 (PMC12221170; doi:10.1371/journal.pone.0325846)
Supplement: S1 File — (PDF) [file pone.0325846.s004.pdf]

	Absorbance			Concentration				
Sample ID	1	2	3	1	2	3	Average Conc.	Stdev
1a	0.214	0.213	0.214	58.46558875	58.21722177	58.46558875	58.38279976	0.143394747
1c	0.307	0.304	0.306	81.56371855	80.81861759	81.31535156	81.23256257	0.379386839
2a	0.303	0.302	0.304	80.5702506	80.32188362	80.81861759	80.5702506	0.248366987
3a	0.178	0.176	0.176	49.52437722	49.02764325	49.02764325	49.19322124	0.286789494
3c	0.315	0.315	0.313	83.55065445	83.55065445	83.05392047	83.38507646	0.286789494
4a	0.172	0.173	0.171	48.0341753	48.28254228	47.78580831	48.0341753	0.248366987
4c	0.237	0.234	0.235	64.17802946	63.4329285	63.68129548	63.76408448	0.379386839
5a	0.233	0.231	0.232	63.18456151	62.68782753	62.93619452	62.93619452	0.248366987
6a	0.227	0.225	0.227	61.69435959	61.19762561	61.69435959	61.52878159	0.286789494
7a	0.213	0.212	0.212	58.21722177	57.96885478	57.96885478	58.05164378	0.143394747
8a	0.16	0.162	0.161	45.05377145	45.55050543	45.30213844	45.30213844	0.248366987
9a	0.168	0.166	0.165	47.04070735	46.54397338	46.29560639	46.62676237	0.379386839
9c	0.221	0.22	0.221	60.20415766	59.95579068	60.20415766	60.12136867	0.143394747
10a	0.247	0.246	0.245	66.66169933	66.41333234	66.16496535	66.41333234	0.248366987
11a	0.176	0.174	0.176	49.02764325	48.53090927	49.02764325	48.86206525	0.286789494
11c	0.171	0.17	0.171	47.78580831	47.53744132	47.78580831	47.70301931	0.143394747
12a	0.213	0.211	0.211	58.21722177	57.72048779	57.72048779	57.88606578	0.286789494
